# Practitioner and Instructor

**Published:** 1885-04

**Authors:** C. E. H. Phillips

**Affiliations:** New York


### PRACTITIONER AND INSTRUCTOR.

BY C. E. H. PHILLIPS, D. D. S., NEW YORK.

Every practitioner of dentistry should be a teacher as well; not alone in the instruction to his patients as to the care of their teeth and surrounding tissues, and in the proper use of the brush, but carefully to impress upon parents the great importance of giving early attention to their children's teeth, though they be "only the temporary ones."

Upon this subject there is surprising ignorance. Almost daily are such questions asked as "Doctor, do you advise filling temporary teeth?" or (with marked surprise), "A friend of mine has had her child's first teeth filled by her dentist—is this proper treatment?" When in reply it is stated how *very proper* such treatment is, and why, the surprise is not diminished, and one is almost sure to have a little patient added to his list, with the self-consciousness of having prevented much suffering to child and annoyance to parent.

Few parents appreciate the value of deciduous teeth, and the family physician heretofore has too often advised their extraction as soon as they became troublesome. The intelligent physician today, recognizing dentistry as a specialty of medicine, when these little sufferers come to his notice sends them to the dentist for treatment, and teeth are now saved instead of being sacrificed, as was formerly the case.

Some patients imagine that they carefully inspect their children's teeth, and do it frequently. This is not sufficient, as irregularity may take place, or decay form in localities capable of being detected only by the glass and instruments, in hands schooled to their use.

Advice imparted to those having children in their care as to the importance of an early visit to the dentist, will be gratefully received. It should be urged not only to prevent pain, which is likely to follow decay, but that these teeth, though "only temporary," should be retained in good condition for masticatory purposes, thereby bettering the general health. When this and the intimate relation the deciduous teeth bear to the permanent ones (the germs of which are at the same time present), and the development of the maxillary bones consequent upon their retention have been made known to patients, then, and then only, has the dentist's duty been performed.

